# Idiopathic Harlequin Syndrome in a Patient from Ethiopia: A Case Report

**DOI:** 10.4314/ejhs.v33i2.24

**Published:** 2023-03

**Authors:** Dereje Melka, Mehila Zebenigus

**Affiliations:** 1 Department of Neurology, Addis Ababa University School of Medicine, Addis Ababa, Ethiopia; 2 Yehulshet Neurology specialty Clinic, Addis Ababa, Ethiopia

**Keywords:** Idiopathic Harlequin syndrome, case report, Ethiopia

## Abstract

**Background:**

Harlequin syndrome is a rare disorder caused by autonomic nervous system dysfunction. It manifests as asymmetric facial flashing and sweating with contralateral anhidrosis. It may be primary (idiopathic) with a benign course or can occur secondary to structural abnormalities or iatrogenic factors. To our knowledge, there has been no report of idiopathic Harlequin syndrome published from Ethiopia. We are reporting this case since it signifies the existence of idiopathic Harlequin syndrome in our setting and the need to properly diagnose this condition.

**Case Presentation:**

We are reporting a 29-year-old female from Addis Ababa, Ethiopia, who presented with a complaint of left hemifacial hyperhidrosis of 8 years which worsen after routine household activities and exercise. Physical examination revealed left hemifacial hyperhidrosis with right-side anhidrosis. She was diagnosed with idiopathic Harlequin syndrome after an appropriate investigation revealed a nonremarkable finding. Symptomatic treatment showed no significant improvement and the patient was also counseled on the disease entity.

**Conclusions:**

The patient described here signifies an idiopathic Harlequin syndrome in an Ethiopian woman. This case highlights the existence of idiopathic Harlequin syndrome within our setting and the need to properly diagnose this condition.

## Introduction

Harlequin syndrome is a rare disorder caused by autonomic nervous system dysfunction. It manifests as asymmetric facial flashing and sweating with contralateral anhidrosis in response to exercise, heat, and emotional factors. The syndrome may be primary (idiopathic) with a benign course or can occur secondary to structural abnormalities or iatrogenic factors (1), (2). Although it is frequently acquired, up to 6% of reported cases can be congenital. Some of the secondary causes are Guillain-Barré syndrome, diabetic neuropathy, brainstem infarction, carotid artery dissection, toxic goiter, superior mediastinal neurinoma, syringomyelia, multiple sclerosis, internal jugular vein catheterization, and iatrogenic effects of invasive procedures (3). The exact mechanism underlying idiopathic Harlequin syndrome remains uncertain. It is most commonly seen in females (1). Idiopathic Harlequin syndrome does not require medical or surgical intervention, however sympathectomy or botulinum toxin injection may be indicated if the symptom has a negative social or psychological impact (4).

To our knowledge, there has been no report of Harlequin syndrome published in Ethiopia. Herein, we report the first case of idiopathic Harlequin syndrome in Ethiopia. We are reporting this case since it signifies the existence of idiopathic Harlequin syndrome in our setting and the need to properly diagnose this condition.

## Case Presentation

**History**: The patient is a 29-year-old female from Addis Ababa, Ethiopia. She presented to one of the private clinics in Addis Ababa on April 16, 2012, with a complaint of left hemifacial hyperhidrosis of 8 years which worsen after routine household activities and exercise. Associated with this she also had pain and numbness in the left upper limb. Otherwise, there was no personal or family history of diabetes or other chronic illness. She had no history of surgery, trauma, and skin rash.

**Figure 1:**
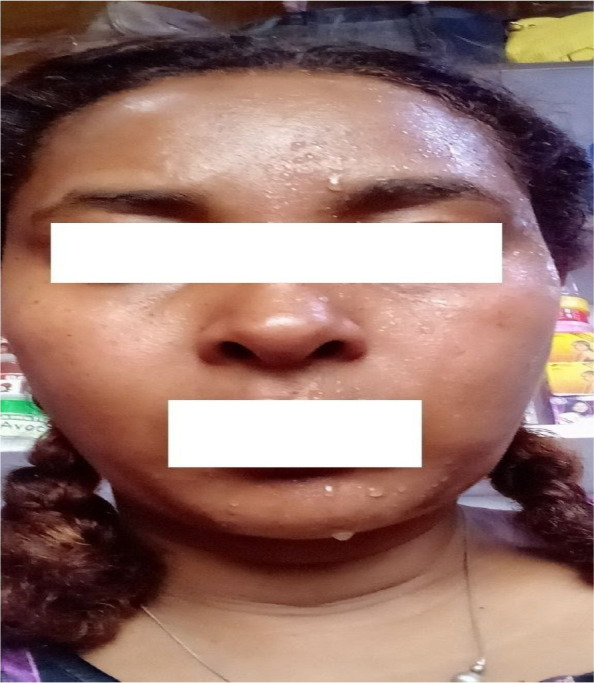
Patient demonstrating feature of Harlequin syndrome: Apparent sweating on the left hemiface with right side anhidrosis

**Physical examination:** The patient's vital signs were normal. The general systemic examinations were normal, and there were no signs of a change in skin color. The patient was alert and oriented with fluent language and intact comprehension. Cranial nerves were normal except for the presence of left hemifacial hyperhidrosis with right hemifacial anhidrosis (Figure 1). Muscle bulk, tone, and muscle power were normal with normal deep tendon reflex. The plantar responses were down-going bilaterally. Sensation was intact for light touch, pinprick, vibration, and position throughout. No coordination abnormalities were detected. Meningeal irritation signs were also negative.

**Auxiliary examinations**: Her complete blood count, erythrocyte sedimentation rate, liver function test, lipid profile, renal function test, and plasma glucose levels were normal. Serum VDRL, Rheumatoid factor, and C-reactive protein results were non-reactive; HBsAg and anti-HCV antibodies and serology for retroviral infections were all negative.

**Figure 2:**
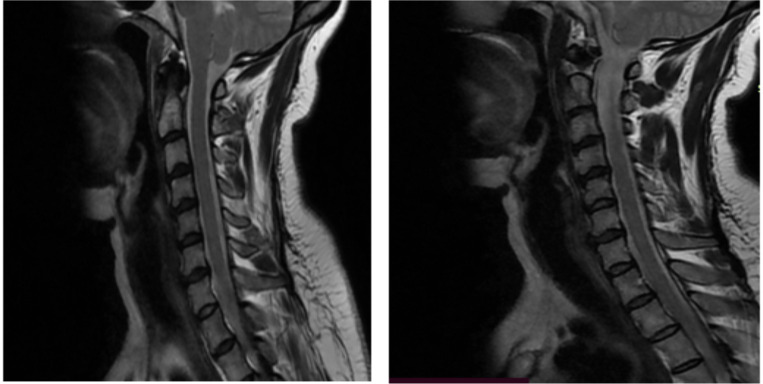
Magnetic resonance imaging studies. Sagittal T2-weighted Magnetic resonance imaging of the cervical and upper thoracic region showed normal finding, except for the presence of C4-5, C5-6, and C6-7 mild anterior thecal indentation by bulged discs

The nerve conduction study of the upper and lower limbs was also normal. The brain MRI result was normal. Cervical and upper thoracic MRI results suggested normal findings, except for the presence of C4-5, C5-6, and C6-7 mild anterior thecal indentation by bulged discs (Figure 2).

**The course of treatment**: She was given Glycopyrrolate topical cream which showed no significant improvement and the patient was counseled on the disease entity. The patient was on follow-up for 10 years with no significant worsening or improvement of symptoms.

## Discussion

To our knowledge, there have been no cases of idiopathic Harlequin syndrome reported from Ethiopia. This case shows the existence of idiopathic Harlequin syndrome occurring in Ethiopian patients.

Due to the rarity (3) and low prevalence of such disease, there will be low expectation of this disease in our setting often leading to misdiagnosis.

Horner syndrome may mimic some of the symptoms and signs of harlequin syndrome, but our patient doesn't have ptosis and miosis which makes Horner syndrome unlikely.

Although idiopathic Harlequin syndrome is usually benign (1), physicians should consider performing a detailed clinical assessment to rule out underlining secondary causes.

Since Harlequin syndrome may be primary (idiopathic) or secondary (1), differentiating this is very important to decide on appropriate and timely treatment for the secondary causes. Guillain-Barre syndrome, diabetic neuropathy, brainstem infarction, carotid artery dissection, toxic goiter, superior mediastinal neurinoma, syringomyelia, multiple sclerosis, internal jugular vein catheterization, and iatrogenic effects of invasive procedures are some of the secondary causes. Even if the clinical presentation of primary Harlequin syndrome is similar to the secondary causes, the absence of laboratory/imaging evidence made secondary causes unlikely in our case.

In idiopathic Harlequin syndrome, the prognosis is favorable (5). Medical or surgical treatments are not usually needed (4). However, symptomatic treatment may be indicated when symptoms affect patients' quality of life. One case report indicated successful treatment of the symptom with oxybutynin and propranolol (5). We tried our patient on Glycopyrrolate topical cream but showed no significant improvement. Subsequently, the patient was counseled on the favorable prognosis of the disease course.

In conclusion, idiopathic Harlequin syndrome is a rare disease entity that exists in our setting, and this case emphasizes the importance of accurately diagnosing this disorder. We believe that the awareness created here may be valuable to health professionals.
